# In Vivo Lymphatic Circulating Tumor Cells and Progression of Metastatic Disease

**DOI:** 10.3390/cancers12102866

**Published:** 2020-10-05

**Authors:** Mikyung Han, Julia Alex Watts, Azemat Jamshidi-Parsian, Urooba Nadeem, Mustafa Sarimollaoglu, Eric R. Siegel, Vladimir P. Zharov, Ekaterina I. Galanzha

**Affiliations:** 1 Interdisciplinary Biomedical Sciences, Graduate School, University of Arkansas for Medical Sciences, 4301 W. Markham St., Little Rock, AR 72205, USA; mhan@uams.edu (M.H.); JAWatts@uams.edu (J.A.W.); 2 Department of Radiation Oncology, University of Arkansas for Medical Sciences, 4301 W. Markham St., Little Rock, AR 72205, USA; JamshidiAzema@uams.edu; 3 Department of Pathology, University of Chicago, 5841 S. Maryland Ave., Chicago, IL 60637, USA; Urooba.nadeem@uchospitals.edu; 4 Department of Otolaryngology, University of Arkansas for Medical Sciences, 4301 W. Markham St., Little Rock, AR 72205, USA; MSarimollaoglu@uams.edu (M.S.); ZharovVladimirP@uams.edu (V.P.Z.); 5 Department of Biostatistics, University of Arkansas for Medical Sciences, 4301 W. Markham St., Little Rock, AR 72205, USA; SiegelEricR@uams.edu

**Keywords:** metastasis, marker, circulating tumor cells, lymphatic vessels, lymphography, sentinel lymph node, in vivo flow cytometry, photoacoustics, personalized prognosis

## Abstract

**Simple Summary:**

Deadly metastases occur when tumor cells are shed from primary tumor into lymph and blood that circulate in distinct networks of vessels and disseminate circulating tumor cells (CTCs) through the body. Therefore, detection of CTCs at potentially treatable early disease stage might improve patient survival. However, most efforts have been made to test CTCs in blood only. Here, we explored the clinically relevant photoacoustic and fluorescent flow cytometry for early in vivo detection of lymphatic CTCs using metastatic melanoma and breast cancer mouse models. We demonstrated the presence of detectable lymphatic CTCs at pre-metastatic disease, estimated correlation between CTCs, primary tumor, and metastasis, and observed parallel CTC dissemination by blood and lymph. Our findings suggest the use of lymphatic CTC testing in vivo as a new indicator of metastasis initiation, and combined assessment of two body fluids as a more promising diagnostic platform compared to existing mono-detection of blood CTCs.

**Abstract:**

The dissemination of circulating tumor cells (CTCs) by lymph fluid is one of the key events in the development of tumor metastasis. However, little progress has been made in studying lymphatic CTCs (L-CTCs). Here, we demonstrate the detection of L-CTCs in preclinical mouse models of melanoma and breast cancer using in vivo high-sensitivity photoacoustic and fluorescent flow cytometry. We discovered that L-CTCs are be detected in pre-metastatic disease stage. The smallest primary tumor that shed L-CTCs was measured as 0.094mm×0.094mm, its volume was calculated as 0.0004 mm^3^; and its productivity was estimated as 1 L-CTC per 30 minutes. As the disease progressed, primary tumors continued releasing L-CTCs with certain individual dynamics. The integrated assessment of lymph and blood underlined the parallel dissemination of CTCs at all disease stages. However, the analysis of links between L-CTC counts, blood CTC (B-CTC) counts, primary tumor size and metastasis did not reveal statistically significant correlations, likely due to L-CTC heterogeneity. Altogether, our results showed the feasibility of our diagnostic platform using photoacoustic flow cytometry for preclinical L-CTC research with translational potential. Our findings also demonstrated new insights into lymphatic system involvement in CTC dissemination. They help to lay the scientific foundation for the consideration of L-CTCs as prognostic markers of metastasis and to emphasize the integrative assessment of lymph and blood.

## 1. Introduction

Metastasis arises from individual tumor cells that are shed from the primary lesion into distant organs, usually by way of body fluids, including blood, lymph and cerebrospinal fluid [1,2,3]. In 2004, it was reported that counts of circulating tumor cells (CTCs) in blood samples can serve as the new prognostic criterion for the survival of patients with metastatic breast cancer [4]. However, further experimental and clinical studies of metastatic tumors (e.g., melanoma, breast and prostate cancer) have demonstrated controversial results. Some authors reported the significance of the in vitro counting of blood CTCs (B-CTCs), while other researchers did not find this test worthwhile for two major reasons: firstly, because of the low sensitivity of in vitro tests, and secondly, due to the heterogeneity of the B-CTC population [5,6,7,8,9,10]. To define the B-CTC subpopulation(s) responsible for the initiation and progression of metastasis, researchers implemented multiple molecular, genetic and epigenetic assays [7,8,9]. To increase sensitivity, in vivo flow cytometry was introduced for the testing of potentially almost all of the whole blood volume instead of analyzing small samples in vitro [10,11,12,13,14,15,16]. Preclinical studies from ours and other laboratories using clinically relevant in vivo photoacoustic (PA) flow cytometry (PAFC) platforms and experimental in vivo fluorescent flow cytometry (FFC) demonstrated the ability to count extremely rare and hence early B-CTCs with a sensitivity unachievable by in vitro methods [11]. Specifically, PAFC offers the capability to identify single fast-moving cells (up to 5–10 cm/s) in blood vessels through the skin in vivo. PAFC does this by using high pulse-repetition-rate lasers (up to 0.5 MHz) and a focused ultrasound transducer which are insensitive to light scattering and autofluorescence background [10,11,12,17,18]. As a result, PAFC identifies single cells with endogenous (e.g., melanin in melanoma CTCs) or exogenous (e.g., low toxic gold nanoparticles, spasers, and layered composite structures) PA contrast agents in multicellular flow without skin incision and extraction of blood samples (i.e., noninvasively) [10,11,12,18,19,20,21,22,23]. Furthermore, the high sensitivity of PAFC requires only a small amount of absorbing contrasts inside a cell to make them detectable [18,19,20,23,24]. For example, we demonstrated the successful PAFC detection of weakly absorbing tumor cells labeled with only 20-30 gold nanoparticles per cell in contrast to the thousands (if not millions) of fluorophore molecules required for fluorescence detection [12,24]. This point is highly important for in vivo application because it reduces toxicity concerns. In addition, similar to the conventional flow cytometry, PAFC can operate in multicolor mode. This allows for the detection of heterogeneous CTCs based on the identification of several receptors by using multicolor nanoparticles conjugated with different molecules specific to CTCs such as EpCam and Folate for breast cancer CTCs [10,12,18]. Another tremendous benefit of PAFC is its successful use in clinical trials [17]. In melanoma patients, we demonstrated that the B-CTC PAFC-based test in vivo has ~1000-times higher sensitivity than available tests in vitro [17].

These achievements have contributed to progress in understanding B-CTCs. However, we still cannot control and stop CTC dissemination, and patients with metastatic disease continue to die every day [25]. One of the major gaps in our knowledge is our lack of understanding of lymphatic CTCs (L-CTCs). It is well established that many primary tumors shed cells into the intra-tumoral and peri-tumoral lymphatic vessels. These L-CTCs travel with unidirectional lymph flow through distinct networks of pre-nodal (afferent) lymphatic vessels toward the nearest sentinel lymph node (SLN), which is, potentially, the first metastatic site [26,27,28,29,30]. Although the presence of tumor cells in the SLN is used as a key parameter for staging tumor and metastasis progression [31], the role of afferent L-CTCs before they colonize SLN(s) remains almost unexplored. Being able to detect them before they colonize the SLN would help us to better understand the metastatic cascade and to diagnose and predict metastatic disease. 

The major obstacle to detecting afferent L-CTCs is that lymph is a challenging substance to study. Compared to blood vasculature, lymphatic vessels are tiny, colorless structures with relatively low pressure and low cell concentration [32,33]. Therefore, finding lymphatic vessels requires additional labeling and mapping using lymphography [34,35,36]. Moreover, lymph sampling is not currently routinely performed in clinical practice. The collection of lymph samples is tedious and challenging. It requires long-term cannulation and yields only a few microliters of lymph [37,38]. These factors make classical assays for screening cells in vitro (e.g., conventional flow cytometry, PCR, genomic and proteomic tests) difficult for L-CTC analysis. A few in vivo studies have detected tumor cells in lymphatic vessels using intravital fluorescent optical microscopy [39], but this approach requires open surgery and misses the identification of relatively fast moving cells.

**Figure 1:**
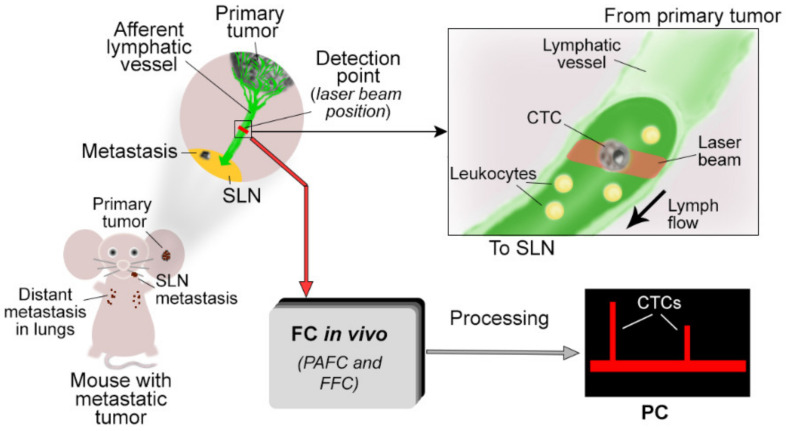
Design of the lymphatics circulating tumor cell (L-CTC0 detection in preclinical animal models of metastatic tumors in vivo.

Taking into account the aforementioned advantages of in vivo flow cytometry for counting B-CTCs, this diagnostic platform seems highly favorable to assess L-CTCs. However, in vivo FFC has never been used for monitoring L-CTCs. PAFC has demonstrated success and a capability to detect L-CTCs with the unprecedented sensitivity of one L-CTC in the background of 10^6^ white blood cells [36], but it is still not a well-developed approach for L-CTC research. Here, we used mouse melanoma and breast cancer models and an advanced platform of PAFC and FFC to demonstrate their capability for preclinical lymphatic research in vivo with a focus on the detection of L-CTCs in early stages of tumor development (Figure 1).

## 2. Results

### 2.1. Optimization of PAFC and FFC Platforms for Detection of L-CTCs

To count L-CTCs, we integrated the technical platforms of PAFC and FFC in vivo. In our previous studies, we defined the optimal parameters of both PAFC and FFC, and evidenced that melanoma CTCs creates well-distinguished PA signals above the background of lymph and blood using natural melanin inside melanoma cells as high-contrast intrinsic PA agents [10,11,17,18,35,36]. Based on the absorption spectra of melanin, its PA signal is maximal in the visible-wavelength range. In the near-infrared range, PA signal from melanin decreases but remains still well-detectable with PAFC [11]. However, in comparison to visible light, near-infrared light has important benefits for the detection of melanoma cells in vivo, namely its low scattering in biotissues, which reduces background noise and provides deep penetration in tissue [10,18]. Furthermore, near-infrared absorption of melanin at 800–850 nm is much higher than the absorption of hemoglobin; this reduces possible false signals from rare erythrocytes that can be found in afferent lymph. Our experience with PAFC confirmed that melanoma cells can be distinguished from erythrocytes in mouse blood vessels at 820 nm [11]. 

The next important step was the selection of a contrast agent for lymphography. Among different lymphographic agents such as Evans Blue (EB), Indocyanine Green (ICG) and gold nanoparticles, we chose EB dye because its absorption is within the visible and far-red spectral ranges (≤690 nm) and it does not interfere with the near-infrared region (e.g., 820 nm) used for the PA detection of melanin in L-CTCs [35,36]. 

To detect low-absorbance L-CTCs (e.g., from breast cancer), we genetically encoded them with fluorescent proteins (e.g., Dendra2) before inoculating them into mice, and counted them in vivo with FFC at an appropriate wavelength. Specifically, from our experience with the detection of Dendra2-encoded carcinoma B-CTCs, we used a continuous wave (CW) laser at the wavelength of 488 nm, which excites Dendra2’s green fluorescence (i.e., excitation, 488 nm; emission, 507 nm) [40]. 

Based on the aforementioned considerations, we chose in this study to use PAFC at 820 nm (energy fluence, 140 mJ/cm^2^), FFC at 488 nm (power, 5 mW) and lymphography with EB contrast agents. The laser parameters were considered as almost optimal because they do not alter vascular function and provide a detection of >90% of CTCs [10,11,18,40]. 

**Figure 2:**
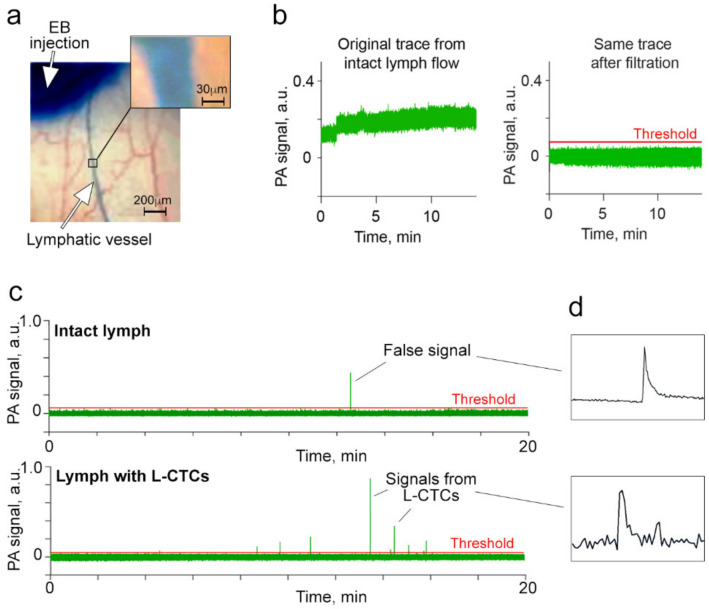
Optimization of photoacoustic (PA) flow cytometry (PAFC) to assess L-CTCs in mice in vivo. (**a**) Evans Blue (EB) lymphography of ear afferent lymphatic vessels. (**b**) The PA trace recorded from the intact (control) lymph without false signals before (left) and after (right) high-pass filtration. (**c**) The PA trace recorded from the intact (control) lymph with one false signal and from the lymph with melanoma L-CTCs. (**d**) The representative peak shapes from PA traces identified as artifact-associated false signal (top) and CTC-associated signal (bottom). The examples of eight independent experiments of control mice and five melanoma-bearing mice are shown in b–e.

To build a signal-processing algorithm, we used healthy mice for PAFC (first control group, *n* = 8) and FFC (second control group, *n* = 3), and training group of melanoma-bearing mice (*n* = 5). To estimate background and false signals, afferent lymphatic vessels in the ear of control and training mice were monitored for 30 min after EB-based lymphography (Figure 2a). 

Then, recorded PAFC and FFC traces were analyzed in four steps. First, we high-pass filtered the recorded PAFC and FFC traces (see Section 4.3. for details) to remove background fluctuations due to breathing and unexpected movements of mice and eventually to select the threshold (Figure 2b) [17,40]. 

We found that most of the control mice (62.5%) did not exhibit any PA signals above the selected threshold (Figure 2b) while 37.5 % of mice showed 1–2 false signals only (Figure 2c, top). The average false-positive rate in the intact lymph of control mice was estimated as 0.3 peaks per 30 min, which we subtracted from groups of melanoma-bearing mice in our second step. 

Moreover, we compared the shapes of false signals from control mice (Figure 2c,d, top) and PA signals from mice with melanoma, which is likely associated with CTCs (Figure 2c,d, bottom). This analysis allowed us to distinguish CTC-related signals (Figure 2d, bottom) from false artifact-related (electrical, vibration and etc.) signals (Figure 2d, top). Therefore, in our third step, we used signal shape as the additional criterion to define true CTC-related signals. 

Our fourth step in the analysis of each trace consisted simply of counting CTC-associated signals per 30 min of monitoring. 

### 2.2. Growth of Primary Melanoma 

**Figure 3:**
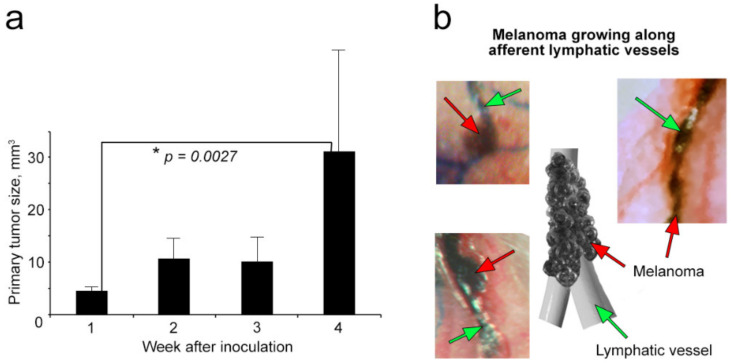
Progress of primary melanoma in the mouse ear. (**a**) Primary tumor size (mean ± standard error of the mean (SEM)). (**b**) Satellite lesions growing along the afferent lymphatic vessels, connecting primary tumor and sentinel lymph node (SLN).

To study metastatic disease, we primarily focused on melanoma because (1) melanoma is one of the most aggressive metastatic tumors in humans, (2) current therapy often has low efficacy, (3) multiple clinical observations indicate that melanoma uses the lymphatic system to metastasize, and (4) melanoma CTCs provide well-readable PA signals in label-free mode [25,30,35,36,41,42]. The preclinical model of melanoma was developed through inoculation of tumor cells (B16-F10 cell line) into the mouse ear. Primary tumor growth was intravitally monitored over four weeks (Figure 3a). To quantify tumor growth, we measured its 2D size with a caliper and calculated its 3D volume in the manner previously reported [43]. 

We determined that all mice had small primary tumors with volumes whose average ± SEM was 4.63 ± 1.06 mm^3^ at week 1 after inoculation. Over the next three weeks, tumors progressively grew with well-defined individual dynamics (Figure 3a). In some mice, we also observed satellite tumors near the primary lesion, including melanoma growing along afferent lymphatic vessels (Figure 3b). 

### 2.3. Progress of Metastasis: Histological and Immunohistochemical Analysis

**Figure 4:**
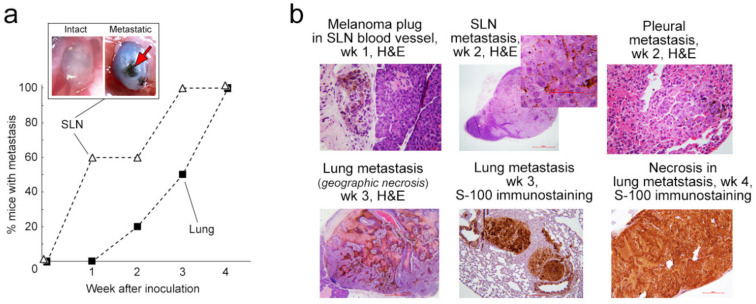
Metastatic disease in melanoma-bearing mice. (**a**) Percentage of mice with histologically detectable SLN and distant metastasis; inset: intravital photos of intact (left) and metastatic (right; week 2 after inoculation) cervical lymph node. (**b**) Histology and immunohistochemistry of organs from mice bearing ear melanoma.

At the end of weeks 1, 2, 3, and 4, a group of mice was sacrificed for histological examination of the metastases present in SLNs and distant organs. At week 1 after inoculation, 40% of melanoma-bearing mice did not exhibit any histological signs of metastases in SLNs or distant organs (similar to clinical stage N_0_M_0,_ Figure 4a). Other mice demonstrated small clusters of tumor nodules in the SLN that took up an average ± SEM of only 9.95 ± 2.72% of its volume (similar to clinical stage N_1_M_0_, Table 1). In a few mice, we also observed small tumor plugs in the blood vessels (Figure 4b). 

During tumor development, subcapsular melanoma deposits in the SLNs subsequently increased in size (week 2 after inoculation, Figure 4a,b) and entirely replaced the SLN by the end of the third week. After the end of week 4, the histologic sections showed SLN metastatic tumors with extracapsular extension and surrounding soft tissue tumor deposits. The metastatic tumor foci showed a higher number of mitotic figures, and the area of necrosis and karyorrhectic debris in the foci increased as the time since inoculation elapsed (Table 1). 

Histological examination of lungs, liver, heart, and brain showed no distant metastasis in any mouse at week 1 (Figure 4a, Table 2). By week 3, distant metastases were present in the lungs (similar to clinical stage M_1_-M_2_) and progressed over the next week, which included an increase in their size, number, mitotic activity, necrosis and karyorrhexis of the nuclei in the metastatic foci (Figure 4b). No metastatic foci were identified in the histologic sections of the brain, heart, and liver at any timepoint. S-100 immunostaining was done to support the melanocytic origin and to highlight the extent of the metastatic lesion (Figure 4b, left and right in bottom row). 

### 2.4. Real-Time Quantification and Individual Dynamic of Spontaneous L-CTCs over Melanoma Progression

To count melanoma L-CTCs, afferent lymphatic vessels connecting primary tumors and SLNs in melanoma-bearing mice were monitored using PAFC for 30 min. PAFC was followed by EB lymphography. The average diameter ± SEM of the examined vessels was 52.1 ± 3.55 μm. 

**Figure 5:**
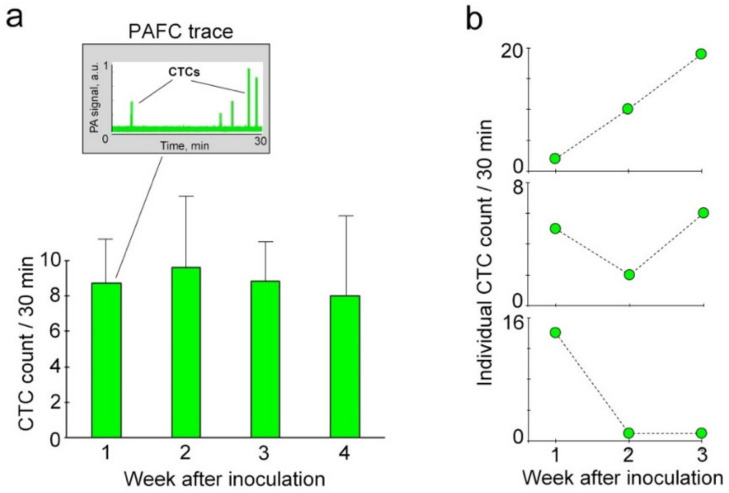
In vivo monitoring of L-CTCs over melanoma progression in groups of mice at week 1 (*n* = 33), week 2 (*n* = 21), week 3 (*n* = 15) and week 4 (*n* = 6) after inoculation. (**a**) Average counts of L-CTCs (mean ± SEM), as determined by PAFC; inset: 30-min PAFC trace from the afferent lymph in the mouse with early melanoma (week 1 after inoculation). (**b**) Representative examples of individual patterns of L-CTC counts.

First, we focused on studying L-CTCs in pre-metastatic and early metastatic melanoma because this stage of disease is potentially treatable and improvement of its diagnosis is highly demanded. We found that 80% of mice with early melanoma (week 1 after inoculation) demonstrated well-detectable PA signals associated with L-CTCs (Figure 5a; Table S1; Video S1). Their average count ± SEM was 9 ± 2.54 L-CTCs per 30 min of monitoring. It was important that L-CTCs were detected in mice with very small primary tumors and without SLN metastasis (similar to clinical stage N_0_M_0_). At week 1 after inoculation, the smallest primary melanoma was measured as 3.00 mm × 0.27 mm, its volume was calculated as 0.11 mm^3^ (½ × 3 × 0.27^2^ = 0.11), and this tumor was able to shed six L-CTCs per 30 min. 

Clinical and experimental studies have also shown that tumors undergo a series of changes during the course of the disease (tumor evolution or “neoplastic progression”) [2,44]. Therefore, our next step was the monitoring of L-CTC counts over melanoma progression. Surprisingly, the average L-CTC count did not significantly change throughout disease progression (Figure 5a; Table S1). However, we found that half of mice with advanced metastasis stopped shedding L-CTCs. In comparison, L-CTCs were detected in 80% of mice with early melanoma.

To explore individual variability in L-CTCs, we analyzed their counts for each mouse in a group of 7 melanoma-bearing mice over a course of 3 weeks. While all mice exhibited L-CTCs, their dynamics had individual characteristics. We detected a progressive increase in L-CTC counts in 45% of mice (Figure 5b, top). Another 45% of mice demonstrated fluctuating counts of L-CTCs from week to week (Figure 5b, middle). In one mouse, we found the highest number of L-CTCs during the early stage (week 1) followed by a dramatic decrease in their number in later weeks (Figure 5b, bottom).

### 2.5. Parallel Progression of L-CTCs and B-CTCs and Their Cross-Talk In Vivo

**Figure 6:**
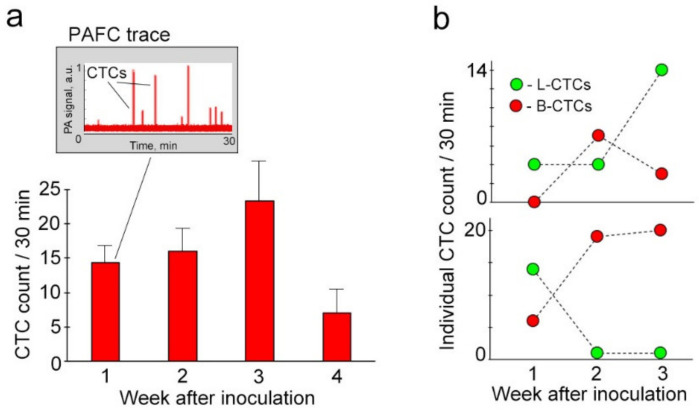
Parallel dissemination of blood CTCs (B-CTCs) and L-CTCs. (**a**) Average counts of B-CTCs (mean ± SEM) in groups of mice at week 1 (*n* = 33), week 2 (*n* = 21), week 3 (*n* = 15) and week 4 (*n* = 6) after inoculation; inset; 30-min PAFC trace from the ear artery in the mouse with early melanoma (week 1 after inoculation). (**b**) Representative examples of individual interrelations between L-CTCs and B-CTCs over disease progression.

To define in vivo cross-correlations between L-CTCs and B-CTCs, ear blood vessels with an average ± SEM diameter of 42.1 ± 4.52 µm were monitored with PAFC in each mouse just after PA assessment of afferent lymphatic vessels (Figure 6). This strategy allowed us to define the relationship between L-CTCs and B-CTCs, and compare the role of lymphatic and blood pathways in CTC dissemination at the same timepoints of disease. Our measurements demonstrated that CTCs commonly used both fluids for dissemination (Figure 5a and Figure 6a). In early melanoma (week 1 after inoculation), both L-CTCs and B-CTCs were detected in the majority (70–80%) of the mice. Approximately 20–30% of mice used only one fluid for CTC dissemination. On average (Figure 6a, Table S1), early melanoma was characterized by non-significant differences between the counts of L-CTCs and B-CTCs: 9 ± 2.54/30 min L-CTCs vs. 14 ± 2.37/30 min B-CTCs at week 1 after inoculation (*p* = 0.11) and 10 ± 4.06/30 min L-CTCs and 16 ± 3.43/30 min B-CTCs (*p =* 0.24) at week 2 after inoculation (*p* = 0.21). As melanoma progressed, we observed some prevalence of B-CTCs over L-CTCs at week 3 (9 ± 2.15/30 min L-CTCs vs. 23 ± 6.48/30 min B-CTCs; *p* = 0.08) and equal counts of L-CTCs and B-CTCs at week 4 (8 ± 4.62/30 min L-CTCs vs. 7 ± 3.24/30 min B-CTCs; *p* = 0.87). The mechanisms of these changes are currently unclear and will be the subject of future studies.

### 2.6. Correlations of L-CTCs Counts with Primary Tumor Size, Metastasis and B-CTCs

**Figure 7:**
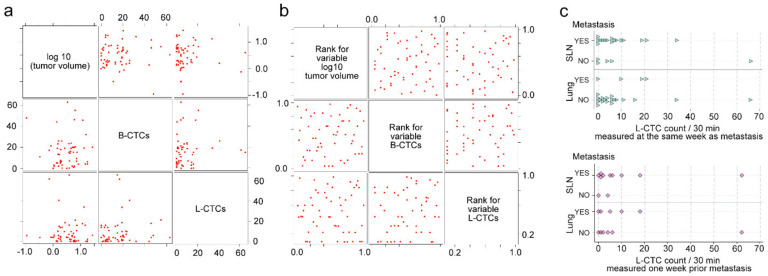
Statistical analysis. (**a**,**b**) Scatterplot matrices of correlation between L-CTC count, B-CTC count and primary tumor volume, *n =* 56 records. (**c**) Scatterplot matrices of correlation between L-CTCs and metastasis.

To analyze the correlations between L-CTCs and primary tumors, tumor volumes and L-CTC counts were plotted over time as spaghetti plots and summarized by observation week as the mean, standard deviation, minimum, median, and maximum (Figure 7a,b). Non-parametric methods for paired data were used to assess the amounts of change in L-CTCs between different weeks of the study. Specifically, for any two weeks to be compared, the paired difference in L-CTC counts was computed and then tested for significant deviation from zero via Wilcoxon’s signed-rank test. Tumor volumes were also log10-transformed to reduce bias due to right-skewing. Then the correlations among log volumes and L-CTCs were quantified using Pearson and Spearman’s correlation coefficients, and visualized using scatterplot matrices. Initially, the correlation analysis did not adjust for the week of observation. To adjust for the week of observation, we computed the weekly means of log volumes and L-CTCs and subtracted these weekly means from their respective data points to obtain mean-centered or “mean-adjusted” values. Then, we repeated the correlation analysis on these mean-adjusted values.

To estimate the possible link between L-CTC and metastasis, we studied correlation for (1) L-CTCs counted just before sacrificing, versus CTCs counted one week earlier and (2) metastases in both the lung and SLN (present/absent) (Figure 7c). For this, we summarized L-CTC counts by the metastatic status group as the group’s mean, standard deviation (SD), SEM, median, and range, visualized them by scatterplots, and compared groups for CTC-count differences with the Wilcoxon rank-sum test.

To analyze the difference between B-CTC counts and L-CTC counts, we computed the paired difference between them within each mouse, then tested the paired differences for significant deviation from zero using Wilcoxon’s signed-rank test. Because many mice contributed a paired difference to multiple timepoints, we conducted a separate signed-rank test by week from week 1 to week 4 in order to make sure that each mouse contributed, at most, one paired difference to each test.

In all comparisons, the significance level was set at alpha = 0.10 (i.e., at *p* < 0.10), which is higher than the usual *p* < 0.05 significance level. Our plan was to consider any comparison that achieved *p* < 0.10 as deserving of a closer look, and to analyze in more detail the group difference and/or the correlation underlying the *p* < 0.10 result. Despite our efforts, we did not find any correlations and comparisons that achieved *p* < 0.10.

### 2.7. Detection of L-CTCs in Metastatic Breast Cancer

Finally, we explored whether early dissemination of L-CTCs is a common phenomenon of mesenchymal (e.g., melanoma) and epithelial tumors. This goal can be achieved by combined monitoring of primary tumor size and CTCs in vivo. However, in contrast to melanoma, epithelial cancers (e.g., breast cancer) do not have endogenous contrast agents, and require additional labeling to identify them in vivo. Among various fluorescent (e.g., proteins and dyes) and photoacoustic (e.g., nanoparticles) exogenous contrast agents, only fluorescent proteins (e.g., Dendra2) are genetically produced in cells during their proliferation and tumor growth inside the body. In turn, the detection of fluorescent CTCs requires the use of the fluorescence module of in vivo flow cytometry (i.e., FFC), which can detect CTCs in only superficial vessels of thin tissues due to strong autofluorescence [13,15,16,40]. Altogether, these considerations suggested that the ear model was optimal.

**Figure 8:**
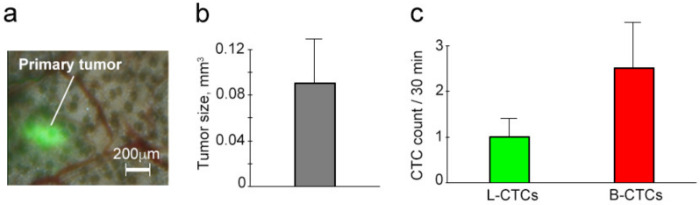
Assessment of mice with early breast cancer. (**a**) Integrated transmission and fluorescence image of primary breast cancer in the mouse ear. (**b**) Primary tumor size. (**c**) The average counts of L-CTCs in afferent lymphatic vessels and B-CTCs in ear blood vessels. Results in “b” and “c” panels are presented as means ± SEM.

To model metastatic epithelial cancer, we inoculated human breast cancer cells (MDA-MB-231 cell line) into mouse ear. To detect optically transparent breast cancer cells, they were genetically encoded with Dendra2 fluorescent protein, which has bright green fluorescence [45]. This allowed us to monitor primary tumor size with fluorescence microscopy and count L-CTCs with in vivo FFC using parameters that were previously established and reported for the detection of carcinoma cells with Dendra2 in blood vessels [40]. Similar to the study of melanoma CTCs, the design of the experiment involves sequential FFC monitoring of afferent lymphatic vessels (30-min) and blood vessels (30 min). We observed that primary breast cancer grew well in the ears of 75% of mice 2 weeks after inoculation (*n =* 9) (Figure 8a,b).

At week 3 after inoculation, tumors started to regress, and they disappeared over the next couple of weeks. To obtain reliable results, we used only data that were received before tumors started to regress. Despite our model being only partly successful, it nonetheless allowed us to study CTCs at the earliest occurrence of breast cancer (2 weeks after inoculation) with primary tumor volume 0.09 ± 0.04 mm^3^ and without metastasis. In FFC traces at this stage of disease, we obtained extremely rare but well-detectable signals associated with both L-CTCs and B-CTCs (Figure 8c). Individual analyses of mice showed three patterns of dissemination: (1) L-CTCs and B-CTCs; (2) L-CTCs only and (3) B-CTCs only. This was similar to what was observed in melanoma-bearing mice. The most interesting finding was that a primary breast cancer with a volume of only 0.0004 mm^3^ (½ × 0.094mm × 0.094mm^2^ = 0.0004) was able to release L-CTCs (1 L-CTC/30 min).

## 3. Discussion

The capability to intervene in CTC dissemination for the purpose of stopping tumor metastasis is the ultimate goal of experimental and clinical oncology [1,2,3]. However, despite substantial efforts to understand CTC behavior and define diagnostic and therapeutic targets, metastases currently lead to most cancer deaths [25]. The major obstacle is our limited understanding of how CTCs disseminate through the lymphatic system, especially at an early stage of tumor development. The solution to this biomedical problem is challenging. In contrast to blood tests, afferent lymph sampling is currently impractical, and well-established in vivo methods (e.g., MRI, PET, ultrasound imaging) cannot detect individual fast-moving cells in lymph flow [46]. In our early studies, we solved this problem by using an advanced platform of in vivo high-speed PAFC [35,36]. In this study, we successfully used PAFC to count unlabeled L-CTCs in mice with metastatic melanoma using natural melanin as an intrinsic PA contrast agent.

For the first time, to the best of our knowledge, we also showed that L-CTCs with fluorescent tags are easily detectable in vivo using FFC. This new application of in vivo FFC, in experimental medicine and biology, provides the possibility of monitoring fast-moving lymphatic cells in real time in vivo without invasive skin-and-vessel incisions. The successful detection of lymphatic cells genetically encoded with photoswitchable proteins (e.g., Dendra2) would be further used to track single L-CTCs and understand their interplay with host immune cells inside the body that are suggested to be important [47]. Despite its contribution, FFC has considerable limitations for a translation to humans, including (1) low detection depth due to light scattering and the autofluorescent background that prevents the assessment of the majority of human vessels, (2) the potential cytotoxicity of most existing fluorescent labels and (3) the obvious impossibility of transfecting human cells with fluorescent proteins in vivo [13,15,16]. However these problems seem to be solvable by using low-toxicity near-infrared fluorescent tags such as ICG, which is currently acceptable for lymphography in humans, or by using patient-derived xenograft (PDX) models, in which immunocompromised mice are inoculated by specimens from patients with the aim of recapitulating the host’s disease at the organismal, genomic, and epigenomic levels [34,48].

To achieve some parallels between obtained experimental results and clinical scenarios, we used mouse melanoma with spontaneous metastatic disease. The main benefit of this model compared to the colonization of organs through forced injection of tumor cells in circulation [2,49] is the replication of real metastatic disease. This allowed us to study the natural shedding of L-CTCs from the primary tumor and correlate CTC count with the severity of metastasis and tumor growth.

In melanoma-bearing mice, we used well-established determinants of disease progression (e.g., primary tumor size, B-CTC count, and presence of metastasis in SLN and distant organs) and determined the timepoints of metastatic disease that replicated metastatic disease in humans at clinical stages N_0_M_0_ (absence of overt metastases), N_1_M_0_ (metastases in SLN and absence of distant metastasis) and M_1_-M_2_ (distant metastasis) [31]. Our most interesting finding is that melanoma development was sometimes associated with tumors growing along lymphatic vessels near the primary tumor, which is in line with the reported ability of melanoma to develop satellite tumors [50]. The diagnostic value of this phenomenon is currently not clear and requires future exploration.

In terms of the mechanisms of lymphatic metastasis and the physiology of the lymphatic system, the significant impact on the prediction of metastases and the prognosis of their early progression is expected to come from the identification of L-CTCs in early pre-metastatic disease (i.e., before colonization of the SLN) [26,28,29]. Furthermore, the unidirectional character of lymph flow from the primary tumor to the SLN [32] suggests that an afferent L-CTC count reflects the productivity of the primary tumor to shed tumor cells into the lymph. These data encouraged us to pay special attention to our analysis of the earliest L-CTCs. Using highly sensitive in vivo flow cytometry, we found that both melanoma and breast cancer in the pre-metastatic stage (similar to clinical stage N_0_M_0_) were able to release tumor cells into the lymph flow. The productivity of 0.1 mm^3^ primary melanoma and 0.0004 mm^3^ (~0.08 mm in linear scale) breast cancer achieved six L-CTCs and one L-CTC per 30 min, correspondingly. Considering the reported number of cells in a primary tumor (>10^9^ cells in 1cm^3^ tumor [51]), we estimated that 0.0004 mm^3^ breast cancer contains >400 cells and releases ~48 L-CTCs per day while 0.1 mm^3^ primary melanoma contains >1 × 10^5^ cells and sheds ~288 L-CTCs per day and delivers them into an SLN. Indeed, only rare CTCs (0.0001 – 0.1 %) proliferate and form metastasis, but the capability of a single melanoma cell to generate metastasis and the continuous entry of L-CTCs into SLNs support the possibility of metastasis development from those early L-CTCs and, thus, the parallel progression of primary tumor and metastasis [3,52]. Since a clinically detectable primary tumor has a size of ≥1cm^3^ (> 10^9^ cells) [51], our data suggest that lymphatic-related metastasis might be presented at the time of initial diagnosis. Moreover, our results demonstrated that finding L-CTCs at the pre-metastatic stage of disease (e.g., tumor in situ) might serve as the indicator of metastasis initiation.

The early shedding of L-CTCs in both tumor models suggests that the early appearance of tumor cells in afferent lymph is an attribute of mesenchymal and epithelial tumors [3]. Based on the traditional considerations that the capacity of tumor cells to invade vessels increases as the primary tumor grows [2], we would have found an increased number of L-CTCs during disease progression. However, our experimental results demonstrated that counts of L-CTCs at early and advanced stages of melanoma were similar, and ~50% of advanced tumors stopped the production of L-CTCs. This can be explained by the fact that as a tumor grows, 50–60% of its volume becomes hypoxic or anoxic. As a result, the ratio of the vascular space to the total tumor mass progressively decreases [53], which may reduce or stop the production of L-CTCs.

The next important question was about the link between bulk L-CTC counts and metastasis progression. Our extended statistical analysis did not show significant correlations between L-CTC count, primary tumor size, existing SLN metastasis and further distant metastasis. The lack of statistical significance might be partly explained by the variability in L-CTC counts and their individual fluctuations throughout disease progression. Furthermore, numerous reports have indicated that B-CTCs are a highly heterogeneous cell population, and only a small number of B-CTCs are able to survive and form metastases [1,2,5,6,7,8,9,53]. The extrapolation of B-CTC results to L-CTCs assumes the existence of metastasis-initiating subpopulations of L-CTCs that would be defined through future genetic, epigenetic, and functional analyses of L-CTCs.

Another fact, which cannot be ignored, is the interrelationship of L-CTCs and B-CTCs. It was suggested that most of the primary tumors can release cells in both lymph and blood [3,27]. In our study, we produced experimental evidence that CTCs can use both lymph and blood pathways for dissemination at the same timepoint of disease. Furthermore, our individual analysis revealed that there are multiple patterns of CTC dissemination. We observed equivalent counts of L-CTCs and B-CTCs, predominant counts of either L-CTCs or B-CTCs, and the spreading of CTCs by only one fluid. These experimental data alarmed us in that the testing of one body fluid might provide false results, and a blood test might be negative in the actual presence of CTCs in an organism. Thus, integrated detection of CTCs in lymph and blood in vivo has promise and could be more sensitive than the traditional counting of CTCs in blood.

In future, we intend to continue building the foundation for the clinical use of PAFC-based lymphatic tests. Based on the safety of PA technology, and the successful use of PAFC in our clinical trials to detect B-CTCs in melanoma patients with anunprecedented 1000-fold improvement in sensitivity [17], we are positioned to rapidly translate the knowledge gained from our studies into clinical practice, initially by using label-free PAFC detection of melanoma L-CTCs integrated with PA lymphography. The translation of PAFC to other tumors that absorb low levels of light can be achieved by molecular labeling of L-CTCs using low-toxicity, highly absorbing contrast agents such as gold nanoparticles, spasers, layered composite structures (core-shells) and biocompatible natural magnetic nanoparticles [10,12,18,19,20,21,22,23,24]. For molecular labeling, contrast agents should be conjugated with molecules (e.g., antibodies, nanobodies and proteins) specific to CTCs. The ability to specifically target cancer cells with bioconjugated nanoparticles has been demonstrated in vitro and in vivo by our team [10,12,20,23,24]. We took advantage of the high absorption of some nanoparticles and the high sensitivity of PAFC to pioneer nanotechnology-based PAFC, which can detect and count B-CTCs in vivo [12]. Recently, we also demonstrated that the superior candidate for clinical translation is natural magnetic nanoparticles produced by magnetotactic bacteria [23] because these nanoparticles are detectable by PAFC and their toxicity was negligible. L-CTC labeling might be based on the well-known ability of many nanoparticles to enter (within several minutes) into lymphatic vessels quickly after a single injection in tissue [35,54,55].

Thus, we believe that our findings will empower the continuing study of L-CTCs to better understand lymphatic-related mechanisms of metastasis (e.g., role of oxygenation in L-CTC release), and, eventually, to develop new, advanced diagnostic tests and therapeutic strategies for the prevention and cure of metastatic disease. Because dissemination of CTCs by lymph is a key event in cancer progression, L-CTCs may have profound implications as prognostic biomarkers for estimating therapeutic efficacy and providing individualized treatment for a wide spectrum of malignancies.

## 4. Materials and Methods

### 4.1. Tumor Cell Lines

Mouse melanoma B16-F10 and human breast cancer MDA-MB-231 cell lines were obtained from American Type Culture Collection, USA. The Dendra2 reporter plasmid was obtained from Clonetech-Takara (Cat.# 632546, Takara Bio USA, Inc., Mountain View, CA, USA) to transfect breast cancer cells.

Both cell lines were cultured as monolayers in Dulbecco’s Modified Eagle Medium (DMEM; Corning, NY, USA), supplemented with 10% fetal bovine serum (FBS; Atlanta Biological, Flowery Branch, GA) and 100 units/ml penicillin/100 µg/ml streptomycin/2 mM L-glutamine (all Corning). Cells were maintained in culture in a humidified incubator at 37 °C and 5% CO_2_ and sub-cultured twice a week. The viability of the cells was checked with a Trypan Blue test.

### 4.2. Mouse Model

In vivo experiments were done with mice in accordance with protocols approved by the UAMS Institutional Animal Care and Use Committee. Mice (nu/nu; weighing 20–25 g) were purchased from a commercial source. The mice were anesthetized with isoflurane.

To establish primary tumors, mice were inoculated in one of their ears with 0.5 × 10^6^ melanoma cells or with 0.5 × 10^6^ breast cancer cells in 5–10 µl of PBS. Mice were monitored over the course of 4 weeks.

After inoculation, tumor-bearing mice were subjected to a weekly noninvasive (i.e., through skin) in vivo procedure of photoacoustic (for melanoma) or fluorescent (for breast cancer) flow cytometry. The primary goal was to obtain counts of L-CTCs, but, in addition, the B-CTC counts in blood circulation were measured as traditional markers. Each session included the sequential assessment of two vessels: 50–200-µm of afferent lymphatic vessels connecting tumor and SLN (cervical for ear primary tumor [35]) and 50–70-µm of ear blood vessels with a total duration of around 60 min. The assessment of lymph was followed by EB lymphography.

At the same timepoints, to assess the growth of primary melanoma in vivo, the tumor, which was visible as a black spot, was measured with a caliper and magnifying glass (Bel-Art, F378650000/EMD). The primary breast cancer tumor, which has green fluorescence, was measured using a fluorescent microscope at a small magnification (2×–4×). Using 2D measurements (long diameter and short diameter), the tumor volume was calculated as ½ × long diameter × short diameter^2^ [43].

At the end of each week, one group of melanoma-bearing mice (*n =* 5–12) was sacrificed to detect metastasis in SLN(s) and distant organs (lungs, heart, liver, brain) by using histology and immunohistochemistry methods. Intact mice (no tumor) served as controls.

### 4.3. In Vivo PAFC and FFC

The basic principles and technical details of the integrated PAFC and FFC have been described in our previous reports [10,11,12,17,18,24,36,40,43]. Briefly, the integrated technical platform was built on an upright Olympus IX81 microscope (Olympus America, Inc., Central Valley, PA) and equipped with pulsed lasers for PAFC and CW lasers for FFC. In our study, PAFC used a laser (LUCE 820, Bright Solutions) of the following parameters: wavelength, 820 nm; pulse energy, 75 μJ; pulse width, 8 ns; and pulse repetition rate, 10 kHz. FFC used a CW laser diode with a wavelength of 488 nm and 5 mW in the sample (IQ1C45 (488-60) G26, Power Tech., Alexander, AR, USA). To monitor L-CTCs and B-CTCs with both PAFC and FFC, anesthetized mice were placed on the heated microscope stage. An ear vessel (lymph or blood) of interest was noninvasively irradiated by a linear laser beam positioned over the cross section of the vessel.

In PAFC, PA signals were detected by an ultrasound transducer (V323-SU, Panametrics NDT Inc.), which was gently placed over the skin close to the point of detection, and passed through an amplifier (model 5662: bandwidth, 5 MHz; gain, 54 dB; and model 5678: 40 MHz, 60 dB; both from Panametric NDT). Acoustic contact between the tissue and the transducer was facilitated by warm water or ultrasound gel.

In FFC, fluorescent emission passed through bandpass filters (520 ± 15 nm), achromatic lenses, and slits (200 µm × 3 mm) and were detected by a photomultiplier tube (PMT; model R928, socket HC123-01; Hamamatsu, Bridgewater, NJ, USA).

Finally, both PA and fluorescent signals were recorded by a computer equipped with an analog-to-digital converter board (PCI-5152, National Instruments Corp., Austin, TX, USA). The resulting PAFC and FFC traces were displayed on the computer monitor in real time using custom software (LabVIEW; National Instruments Corp., Austin, TX, USA). PA signals from the melanin in melanoma CTCs and fluorescent signals from Dendra2 in breast cancer CTCs were detected as transient peaks in signal traces (Video S1). Further off-line processing of saved traces and the counting of CTC-related peaks were performed in the MATLAB environment (Mathworks Inc. Natick, MA, USA). Specifically, we first high-pass filtered (f_cutoff_ = 10 Hz) the trace to remove fluctuations. We then used the following formula to calculate the peak threshold value:

Threshold = median (trace) + k * IQR (trace)

where IQR is the interquartile range and k is a threshold coefficient.

**Figure 9:**
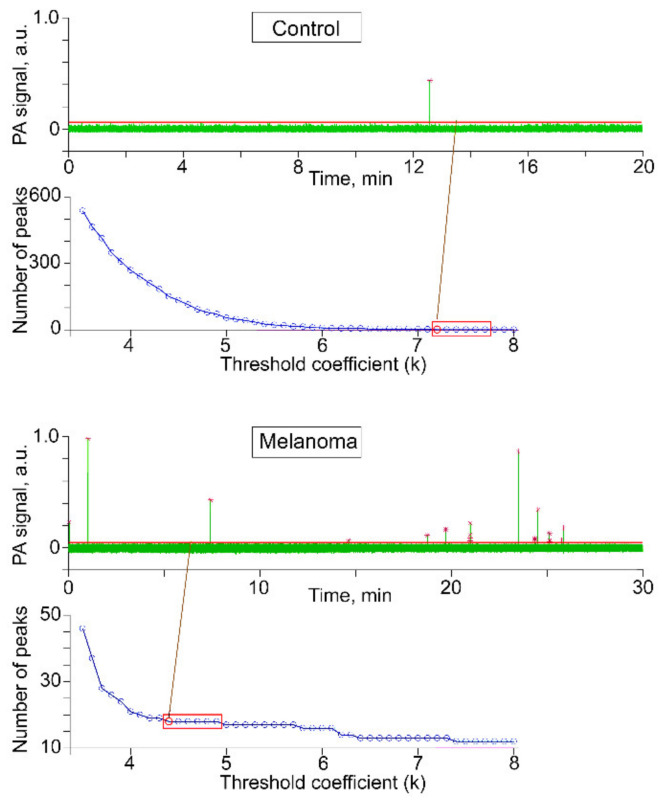
Peak detection threshold selection.

In this step, we selected a range of k values (between 3.5 and 9, with steps of 0.1) and found the number of peaks above the calculated threshold for each value of k (Figure 9). 

As expected, a low k value sets a threshold line inside the envelope of the trace, resulting in too many detected peaks. As k increases, peak count drops quickly. After a certain value of k, peak count is stabilized. When the peak count did not change in six consecutive iterations (Figure 9, **red rectangles**), we selected the first of these points as the threshold coefficient. We used the same algorithm for each experiment.

### 4.4. Mapping Afferent Lymphatic Vessels with Evans Blue (EB) Dye

To visualize rgw optically transparent lymphatic vessels, EB dye (1–3 μl of 1% solution; MP Biomedicals, Inc. Cat. # 151108, MP Biomedicals Inc., Irvine, CA, USA) was injected around the primary tumor. A few minutes after the injection of dye, afferent ear lymphatic vessels became visible as blue channels to the naked eye or by using a magnifying glass.

### 4.5. Histology and Immunohistochemistry

After in vivo monitoring, the primary tumor, SLN, bilateral lungs, heart, liver, and brain were removed. As the B16-F10 cell line is melanotic and hyperpigmented, visual identification of the suspected metastatic lesions could be performed easily. The tissue was placed in 10% neutral buffered formalin for fixation, embedded in paraffin and then processed. The 5–7-mm-thick histologic sections were cut and stained with hematoxylin and eosin (H&E) and evaluated by light microscopy combined with ancillary S-100 immunostaining (polyclonal antibody; Dako; 1/4,000 dilution).

Mitotic activity was determined by counting the number of mitotic figures found in ten consecutive high-power fields (hpf) in the most mitotically active part of the tumor. Only clearly identifiable mitotic figures were counted; apoptotic nuclei and karyorrhectic debris were excluded from this count.

### 4.6. Statistical Analysis

Data for each experiment were summarized by timepoint group as the mean and standard error of the mean (SEM). The CTC results from PAFC and FFC on blood and lymph were converted into CTC rate (number of CTCs per unit of time) in blood and lymph. These estimates were log-transformed and analyzed as the CTC rate ratios in lymph versus blood. For each mouse, the L-CTC rate was expressed as a ratio relative to its B-CTC rate. Rate ratios were log-transformed, and the mean and standard deviation (SD) of the log-transformed CTC concentration ratios were estimated within each tumor-bearing group of mice. The group means were accompanied by 90% margins of error (MEs) calculated from the corresponding subgroup SDs. Then, the subgroup means ±90% MEs on log-transformed ratios were back-transformed to rate ratios and reported as mean ratios with 90% confidence limits.

To provide sufficient material for statistical analysis and ensure robust and unbiased results, we used 3–4 animals or more per group. The differences were considered statistically significant if *p* ≤ 0.05.

## 5. Conclusions

Our study demonstrated that in vivo photoacoustic and fluorescent flow cytometry platform provides highly sensitive diagnosis of L-CTCs with the ability to identify and count the earliest L-CTCs at the pre-metastatic stage of disease (e.g., tumor in situ). 

We obtained experimental evidence that very small primary tumors (currently underdiagnosed) can release tumor cells in lymph fluid, which transport L-CTCs to the SLN before the development of histologically detectable metastasis. 

The generality of early dissemination of L-CTCs was shown for mesenchymal (melanoma) and epithelial (breast cancer) tumors.

Over disease progression, we did not find strong correlations between counts of L-CTCs, metastasis, primary tumor, and stage of disease, possibly due to heterogeneity of L-CTC population. 

Integrated assessment of L-CTCs and B-CTCs demonstrated the parallel spreading of CTCs using lymph and blood fluids. These findings underlined that counting L-CTCs and B-CTCs supplements each other and provides more prognostic information than the blood test alone.

Overall, flow cytometry of lymph fluid in vivo can be broadly applied to various types of tumors to better understand mechanisms of metastasis progression, the complexity of CTC routes, the interplay between L-CTCs and B-CTCs, and to reassess the role of the lymphatic system in the development of metastases.

## Figures and Tables

**Table 1:** Histological detection of SLN metastasis.

Week(s) After Inoculation	Number of Mice	SNL Metastasis				
		Metastasis; % Mice	% SLN Involvement	Mitotic Activity; % Mice	Apoptosis	Necrosis
**1**	N = 12	60	9.95 ± 2.72	No	No	No
2	N = 5	60	63.33 ± 6.67	No	No	Yes
3	N = 10	100	100	11.8 ± 1.1/hpf * (100%)	Yes	Yes
4	N = 5	100	100	13.2 ± 1.3/hpf (100%)	Yes	Yes

Hpf *—high-power fields.

**Table 2:** Histological detection of lung metastasis.

Week(s) After Inoculation	Number of Mice	Distant Metastasis (Lung)			
		Metastasis; % Mice	Mitotic Activity; % Mice	Apoptosis	Necrosis
**1**	N = 12	0	-	-	-
2	N = 5	20	No	No	No
3	N = 10	50	11.7 ± 0.7 / hpf (20%)	Yes	Yes
4	N = 5	100	12.9 ± 0.8 / hpf (60%)	Yes	Yes

## Supplementary Materials

The following are available online at https://www.mdpi.com/2072-6694/12/10/2866/s1, Video S1: Real-time monitoring of afferent lymphatic vessels in melanoma-bearing mouse by PAFC in vivo. Table S1: In vivo L-CTC counts over primary tumor growth and metastasis progression in melanoma.
