# Supplementary material for: In Vivo Lymphatic Circulating Tumor Cells and Progression of Metastatic Disease
Source: Cancers (Basel). 2020 Oct 5;12(10):2866. doi: 10.3390/cancers12102866 (PMC7650582; doi:10.3390/cancers12102866)
Supplement: Supplementary file 1 [file cancers-12-02866-s001.zip › cancers-910278-supplementary-R1/cancers-910278-supplementary.pdf]

Table S1. In vivo L-CTC counts over primary tumor growth and metastasis progression in melanoma.

Week after inoculation	# Mice	L-CTC count/ 30 min, M ± m	B-CTC count / 30 min, M ± m
**1**	33	9 ± 2.54	14 ± 2.54
**2**	21	10 ± 4.06	16 ± 3.43
**3**	15	9 ± 2.15	23 ± 6.48
**4**	6	8 ± 4.62	7 ± 3.24
